# Cancer Prevention Sense Making and Metaphors in Young Women’s Invented Stories

**DOI:** 10.3390/healthcare10112179

**Published:** 2022-10-31

**Authors:** Daniela Lemmo, Maria Luisa Martino, Maria Francesca Freda

**Affiliations:** Department of Humanities, University of Naples Federico II, 80133 Naples, Italy

**Keywords:** breast and cervical cancer screening, young women, invented stories, narrative, metaphors, preventive health care, care processes, psychological clinical intervention

## Abstract

Despite the proven effectiveness of cancer prevention, the literature highlights numerous obstacles to the adoption of screening, even at a young age. In cancer discourse, the metaphor of war is omnipresent and reflects an imperative demand to win the war against disease. From the psychodynamic perspective, the risk of cancer forecasts an emotionally critical experience for which it is important to study mental representations concerning illness and health care. Through the creation of an invented story that offers a framework for imagination, our aim is to understand what the relationship with preventive practices in oncology means for young women and how this relationship is revealed by their metaphors. A total of 58 young women voluntarily participated in the present research, answering a narrative prompt. The stories written by the participants were analyzed using qualitative methodology to identify construct, themes and metaphors. Our findings identify four constructs: the construction of a defense: youth as protection; the attribution of blame about cancer risk; learning from experience as a prevention activator; and from inaccessibility to access to preventive practices: the creation of engagement. The construction of an invented story allows us to promote a process of prefiguration on the bodily, affective and thought planes invested in preventive practice and brings out the use of metaphors to represent cancer risk and self-care. The results allow us to think about the construction of interventions to promote engagement processes in prevention from an early age.

## 1. Introduction

In women younger than 50 years of age, breast cancer is the most prevalent form of cancer, followed by cervical cancer [1,2]. These tumors, in addition to being treatable, with a 5-year survival rate of about 91% for breast cancer and 68% for cervical cancer, are also detectable early or even preventable. In the case of breast cancer, self-examination is recommended for prevention from the age of twenty, in addition to a breast test at least once a year, ultrasound to examine young breasts (30–45 years) and mammography every two years for women aged between 50 and 69 years, which is free of charge as part of health screening programs every two years. Key risk factors are age, familiarity and genetics, as well as the hormonal changes that occur in women during their lifetime. In the case of cervical cancer, in addition to primary prevention through HPV vaccination, screening includes a pap test to detect precancerous cells [3], which is free of charge every three years for the female population aged between 25 and 65 years. The main risk factor for cervical cancer is HPV (human papillomavirus) infection. This virus is transmitted from person to person by sexual or intimate skin-to-skin contact. Having multiple partners, a sexually promiscuous partner or sexual intercourse at an early age, increased the risk of HPV infections, although these causes are necessary but not sufficient for cancer development. 

Despite the proven effectiveness of prevention, the literature highlights numerous obstacles to the adoption of preventive processes. In young women, breast screening appears to be influenced by poor knowledge and awareness of risk factors, warning signs or symptoms and breast self-examination [4,5] Cervical screening may be affected by marital status, age, shame, low socioeconomic status, limited education, lack of access and fear of cervical cancer diagnosis [6,7,8].

According to the psychodynamic perspective [9], talking about cancer prevention brings into play the need to connect to health research, the study of mental representations concerning illness and health care, defense mechanisms and personality aspects connected to health choices [10]. The risk of cancer represents a critical experience for the psyche, which can defend itself with false ideas of integrity and absoluteness, refusing to admit the finitude of existence, therefore sheltering itself from the anguish of death [11,12] and from the traumatic illness experience [13,14,15,16]. In addition, from a gender perspective [17,18], women are encouraged to take care of their bodies, to become protagonists of their own health, to take on this responsibility and to undergo medical examinations in the absence of symptoms, thus exposing themselves to the risk of discovering a sick body [19] in its intimate and unspeakable parts, which symbolize female sexual identity [20,21,22,23]. Regarding young women’s participation in female cancer screening, the most recent literature focuses on the study of interventions aimed at education and prevention, whereas qualitative studies on the narrative construction of a story and the use of metaphors are absent in the field of cancer prevention.

### Inventing Stories: Narration and Metaphors of Health and Illness

The creation of an invented story has a specific function in the natural life of the individual [24,25,26] and offers new dimensions and frameworks for imagination and fantasy. Through such stories, emotional aspects are allowed to access the sphere of consciousness and are processed by imagination, gradually becoming thinkable elements. Invented stories, myths and metaphors belong to the psychic mentality and connect the individual to the great myths of humanity [27] within the sociocultural context, with the function of re-establishing the process of connections as individuals manage difficult personal issues [28]. Invented stories, as a transitional phenomenon [29] during developmental phases, play an organizing role of archaic, non-verbalized elements [30] to find meanings through their narrative sense-making function [31]. Both narratives and metaphors provide mechanisms for making sense of the world. Whereas the latter elaborate and articulate particular points in a narrative, the former provide meaningful connections between sometimes unrelated metaphors, suggesting a relationship between the two [32]. Narration can be considered a model of the functioning of the mind that mediates between subjectivity, otherness and culture [33,34]. Therefore, a story is not a mere succession of events but an organization of events through a process of building links that goes beyond the ostensible aspects of experience, giving shape and place to a process of interpretation of the self, of the world and their report [35]. Here, we define narration as a device of meaning [36] that allows us to elaborate experiences, represent events and think [35], constructing new meanings [37] and transforming the formless into a thinkable form [38]. If, at the basis of every thought activity, there are emotional experiences and sensory impressions that are not elaborated [39], not yet thinkable, people will put into stories what mostly pervades from the proto-emotional point of view [40]. In our approach, unconscious feelings are regarded as lying not outside the realm of language but contained within it; words are considered major symbolic forms. The term symbolic here implies a form of imaginative self-extension from the domain of private experience; metaphorical and symbolic language is the vehicle for the creative expression of personal meaning and associated feelings. Language is used both to pin things down and, symbolically, to create a further dimension. Metaphor is responsible for the sense making and expression of feelings [41], representing their interpretations and thus helping to produce understanding and regulate action; they are processes responsible for the automated and subconscious activation of goals and motives [42,43]. Metaphor analysis can be used to assess the structure and content of the implicit theories responsible for these automated and subconscious processes. By combining and reorganizing abstract and concrete features, metaphors influence thought processes, attitudes, beliefs and actions [44]. Metaphors are an essential element of sense making and a tool to stimulate new understandings and actions [45]. Among various diseases, cancer is the one most often addressed through narrative. It is clearly an extremely difficult experience to translate into language because it is an experience of uncertainty, suffering and fear of dying that interrupts the continuity of existence [46,47]. Unsurprisingly, cancer discourse abounds with metaphors. In particular, in public speeches on cancer, the metaphor of war is omnipresent [48], from society’s “war on cancer” to the individual struggle in “heroic battles” against a “tough enemy” [49,50,51]. Such metaphors reflect an imperative demand to win the “war against disease” [52] and therefore against death itself [53]. There is a standard breast cancer narrative that promotes certain values or ways of experiencing the disease, such as being strong in the face of the disease, fighting and surviving and being positive and optimistic [54,55]. Framing cancer as an enemy appears to have served as an appeal to fear [56,57], which has sometimes proven to be effective for cancer prevention [58] and to increase the perception of cancer risk in young, non-patient populations [59] by encouraging people to “fight” cancer as an enemy. However, such metaphors seem to discourage the experience of those who live in the lack of meaning [60,61]. Few studies [62,63,64] have considered the subjective experience lived by patients in light of the metaphors they use or create. Furthermore, no qualitative studies have examined the metaphors used by women with respect to cancer prevention. Therefore, considering that prevention poses the risk of an early diagnosis of cancer, considering the imaginative power of inventing a story and considering the lack of research on the metaphorical expression of women themselves in the field of prevention, the aim of this work is to understand what the relationship with preventive activity in oncology means for young women and how this relationship is revealed by their metaphors.

## 2. Methods

### 2.1. Participants and Tools

Young women were identified through university groups and contacted by psychologist researchers who, after presenting the objectives and methods of the research project, asked if they were available to participate by inventing a story. The Google Drive module was used, a tool that enables data collection with absolute anonymity, receiving only the content of the response without any reference to the sender. Diffusion among friends and acquaintances of the participants was requested. The inclusion criteria required that participants were under the age of 45. The participation was voluntary; each participant signed an informed consent for their inclusion in the study, as well as a document for the protection of their privacy in accordance with the GDPR EU 2016/679, D.L. 101/2018. The study was conducted in accordance with the Declaration of Helsinki, and approved by the Ethics Committee of the Psychological Research of the Department of Humanities of the University of Naples Federico II (Prot.n. 21/2021).The narrative stimulus was: *“A young woman and the prevention of female tumors”. Invent a story*.

### 2.2. Data Analysis 

The narrative corpus was coded according to a qualitative approach. We used the qualitative method [65] as it allows, starting from written narratives, the development of a theoretical narration concerning the process of construction of meaning triggered by a critical situation, such as that of the early diagnosis of disease or the risk of disease. This method allows researchers to develop a system of categories to organize raw text content into themes derived from recurring concepts according to theoretical constructs. Using this method, we examined each text, including both parts and the whole, performing a top-down and bottom-up analysis. First, we identified the relevant narrative material and then highlighted the recurring concepts. Using a process of progressive abstraction, we grouped these interconnected and recurring concepts and identified the themes that emerged. We considered emerging theoretical constructs as meaning-making processes that approximate the representation of prevention. Secondly, using a bottom-up logic of analysis, we identified trajectories along which the macroprocesses are articulated. Finally, we identified themes for which it was possible to use metaphors in the stories to invent. With respect to analysis of the texts, we performed a comparison involving three independent judges, reaching 93% agreement.

## 3. Results

### 3.1. Subject Characteristics

A total of 58 young women with an average age of 26 residing in Naples (42.2%) and its province (57.8%) participated in the research, inventing a story. A proportion of 15.5% of participants had a high school degree as an educational qualification, 82.8% had a university degree and 1.7% had a middle school diploma. Among them, 63.8% declared that they were engaged or in a stable relationship, 28.2% were single and 8% were married. Among those who were familiar with breast cancer (17 out of 58, or 29.3%), 12 reported that their grandmother had breast cancer, whereas 5 mentioned their aunt. Finally, 20% of participants reported knowing of someone who has had cervical cancer, but among the available options, they indicated “other”.

### 3.2. Constructs, Themes and Metaphors

We identified four theoretical constructs and a number of associated themes and metaphors (see Table 1). The theoretical constructs are as follows: the construction of a defense: youth as protection; the attribution of blame about cancer risk; learning from experience as a prevention activator; and from inaccessibility to access to preventive practices: the creation of an engagement. We classified the themes into three categories: body, affects and thought.

#### 3.2.1. The Construction of a Defense: Youth as Protection

In the narrative analysis, the first construct we identified was the construction of a defense: youth as protection. In inventing a story about prevention, young women symbolically organized a current good health status quo that is supposed to remain unchanged. Faced with the risk of an early diagnosis of the disease, the writers dismissed the experience, underlining the irrelevance of the issue because of their young age.

*Health is invisible and silent; illness makes noise and gives signals.*

This theme emerged from stories in which young women described a life in good health as something that goes almost unnoticed, therefore taking for granted that young people feel good. This theme is classified in the category of the *body*, as if to highlight that in the discourse on prevention, there is a silent body that does not express symptoms to take care of in the absence of visual, tactile and somatic perception of something wrong. The visible–invisible and absence–presence dichotomies related to prevention emerged. Prevention occurs in the absence of visible symptoms. Instead, only the possibility of dealing with what is present and visible in terms of tangible symptoms appears to be representable. For this theme, the metaphor of disease emerged as the extraction of a number or a name; at a young age, the disease is represented as a mere causality or something that can befall anyone.

*The little woman had never really thought about what it meant to take care of herself. Controlling herself in the absence of symptoms seemed exasperating to her, or rather too far from her daily life. She was convinced that, being young and healthy, she did not need it.*

*Being invincible: it can’t happen to me.*

This theme emerged from the stories in which young women expressed the sense of invulnerability going through their stage of life, for example, narcissistic integrity that allows you to keep the anguish of illness at bay. This theme classified in the category of *affects,* showing the activation of defensive mechanisms in which, through the denial of risk, it is possible to maintain an ideal of the self as intact and not vulnerable to any threat.

*Benedetta thinks she is invincible. She believes that what happens to others cannot happen to her too, and so she is confident that nothing can disturb her life. University, family, work, marriage, everything is planned in detail. What can stop her life? She believes she is fit and that nothing could happen to her.*

*Self-care is the aesthetic care of your body.*

This theme emerged from the stories in which young women expressed the sense of care understood as aesthetic care of their physical appearance and therefore as the image of themselves. Diets and plastic surgery are narrated as possible actions to take care of one’s health and appearance, understood as the true frontier of the ego and therefore the scene of the encounter with the other. The theme is classified in the category of *thought*, showing the construction of a meaning around the concept of looking after one’s self. *At the bar with friends, those of a lifetime, in front of a hot chocolate.*

*A small diversion from the strict rules of the diet. The exams to take, the courses to attend, the beautician to book, that little touch to treat oneself, the talk about the usual problems at university.*

#### 3.2.2. The Attribution of Blame about Cancer Risk

In the narrative analysis, the second construct we identified was that of the attribution of guilt in the face of the risk of getting ill. The invention of a story on prevention and therefore the foreshadowing of the risk of early identification of breast and/or cervical cancer opens the question of ‘*why me?*’, which is understood as an urgent need of the mind to be able to find meaning in the events that can break into one’s personal biography by overcoming the defensive barrier of the ego. The stories show how, in trying to find an answer to this question of meaning, young women enter the circle of guilt, used as a scapegoat, a liberating dimension of the risk of the onset of the disease.

*A betrayal located in the body and between generations: breast cancer.*

This theme emerged from stories in which young women narrated breast cancer identified as an enemy growing within them and as a family ghost. In the invented stories, breast cancer originates from inside one’s body, as the result of a poison that enters the body through the air you breathe or the food you eat or even as a disease derived from diseased genes that are handed down from woman to woman in one’s family. This theme is classified in the category of the *body* that, at any moment, can become a sick body—a mortal body that has betrayed and that has not been able to defend itself from internal enemies. For this theme, the following three metaphors emerge: the laceration of the body attacked and filled with poison; the uncontrollable invader; and breast and uterus were twins, and when one got angry, the other tried to reassure it by passing its energy and strength to it. However, sometimes, there was something from the outside that came in and ate bits of both.

*They say genes don’t lie, but Maria claims they frighten. Her grandmother died from breast cancer, at a time when screenings were limited, prevention non-existent. Now she feels betrayed by her own body because dark evil, pain, stress and fear have nestled in her breast making her sick. She is afraid, she asks for help, she asks for love. She is undergoing treatment. She finds herself and goes back to living without fear, loving the freedom of feeling about her.*

*A deceptive and punitive sexuality: cervical cancer.*

This theme emerged from stories in which young women described cervical cancer as a very serious disease, without the possibility of differentiating precancerous states or HPV infections. The cause of the disease is related to the invasion of an external virus linked to sexuality with partners who may have multiple relationships. Additionally, the fear of not being able to have more children emerges, as well as the similarity between the growth of a child in one’s womb and the simultaneous development of the tumor. For this theme, classified in the category of *affects*, narratives were invented by young women who are familiar with breast cancer.

*Once upon a time there was a beautiful and intelligent girl who fell in love with an unscrupulous street boy who, in order to sleep with her, managed to make her believe that theirs could be true love. Once she realized his deception, the young girl saw a gynecologist with the fear that the boy she loved so much could have infected her with some contagious disease, luckily only a trivial one. The girl found a brochure there, in which every step for the prevention of cervical cancer was explained.*

*The difficult transmission of love and self-knowledge.*

This theme emerged from stories in which young women identified little love for themselves and little knowledge of themselves and their bodies as factors stopping them from carrying out preventive practices. In their invented stories, the protagonists narrate the impossibility of taking care of one’s self as a sort of destiny that is passed on from mother to daughter. This theme is classified in the category of *thought*, allowing us to hypothesize an intergenerational transmission of self-love. For this theme, a metaphor emerges about being a woman who stops at the zip of her jeans, as if to highlight the un-namable and unknowable nature of her own genital organs.

*Maybe it’s all of us, our lack of assertiveness, of love for ourselves. Each generation has built its knowledge on sexuality and a consequent preventive form of cancer: my grandmother used ignorant prevention, my mother that of class, my aunt total and indiscriminate prevention, Elena stupid prevention. And I? Which prevention do I want to use?*

#### 3.2.3. Learning from Experience as a Prevention Activator

In narrative analysis, the third construct we identified was that of learning from experience as a preventative activator. It is possible to develop a thought on preventive practices if there is an observational dialogue with previous personal experiences of risk, prevention or disease or with the stories of others. This construct highlights how it seems possible to feed thought and representation through the experience and observation of others, expressing the constructive part of the emulation according to which what others do can be teaching.

*Scars and losses that teach.*

This theme emerged from stories in which young women told of injuries in the body that symbolize losses imagined as related to female cancers, such as breast scarring or mutilation, hair loss, abnormal blood loss and loss of fertility. This theme is classified in the category of the *body* because, imagining the relationship of a young woman with prevention, the teaching to start prevention also seems to be derived from wounds to femininity and losses of femininity that could become concrete and not just symbolic. It is significant that young women who are not familiar with breast cancer concentrated on this theme, evoking the power of the imaginative plane that activates an ideal way to deal with the disease and preventing it.

*The scar will disappear, you will see! Acquaintances and relatives keep saying so. She underwent surgery months ago, but the scar is obviously there, witness on the skin of the recent operation and experience. All discovered by chance, a small abnormal swelling which did not seem serious or troubling. Something transitory, she thought, but to probe any doubt, it was better to carry out some investigation. And then the sudden truth, the discovery: it was a carcinoma. Immediate surgery after a few weeks. The initial fear was great but slowly she became familiar with this new experience. The operation went in the best possible way and the subsequent therapies as well.*

*Stories of illnesses (own or of others) to be transformed into active coping*

This theme emerged from stories in which young women told about their disease, that unexpectedly blocks and makes a woman sick and patient, thus giving her a new identity. The time dimension referred to both the temporality of care and prevention is significant (periodically), both at the time of illness that can come and affect the future. This theme is classified in the category of *affects*, showing how the fear of repetition helps to convert memories into learning by extrapolating from memory an integrative meaning that can represent a life teaching able to play an active role in the relationship with the experience; as a result, autobiographical memories can become learning. For this theme, the metaphor of the waiting room emerges as a merciless purgatory, in which the fear of repetition becomes the expectation of expiation of burdens or not.

*When I learned, 10 years ago, that my mother had breast cancer and had to undergo surgery, perhaps I was not aware of the seriousness of the thing: I was only concerned that the surgery would be done as soon as possible and that it would go well. I was 17 and maybe at that age, you just focus on the present. My mother, on the other hand, told me about it after a few years; one of the first questions she asked the oncologist was this: I have a daughter and a granddaughter. At what age should they begin to check themselves too? My mother knew, but I did not, that young women who are direct relatives of a woman with breast cancer are at risk. Some years ago I started to check myself, and, being prudent, I have in mind the schedule of things to do: my ultrasound and her mammography, my pap-test and hers, my routine blood tests and her second protocol. Today I know that prevention is the best therapy to fight cancer, especially for female patients. So, mom, let’s book and go.*

*The warning of a false alarm or an accident on the way.*

This theme emerged from stories in which young women both expressed false beliefs about the risk of getting sick and invented experiences of false alarms, which guaranteed activation toward preventive practices. This theme is classified in the category of *thought*, highlighting the warning that comes from incidents along the way of one’s development that connect with the importance of prevention.

*Anna, 23, was having a shower when she felt a small ball under her skin in her right breast. Encouraged by her mother, she underwent mammogram which turned out to be a false alarm. Since then, she has repeated the exam every year.*

#### 3.2.4. From Inaccessibility to Access to Preventive Practices: The Creation of Engagement

In the narrative analysis, the fourth construct we identified was one that shows the possible passage from inaccessibility to access to preventive practices: the creation of engagement. The construct expresses the continuum that argues for non-prevention and possible prevention, putting in place attributions of meaning that make prevention possible or impossible: on the one hand, the negative foreshadowing of harmful elements that block the possibility of prevention; on the other hand, the possible resources.

*Prevention that creates irreparable damage.*

This theme emerged from stories in which young women invented a kind of paradox whereby prevention itself can create harm rather than protect. If prevention involves the risk of discovering a bad disease, the impediments that may arise are put on the field both in terms of maternity and of damage to the image of one’s own body. This theme is classified in the category of the *body*, which presents itself as confused in its parts.

*It is always thought that it can never happen to young women, that a tumor can never harm them. But one day, after years of using birth control pills, she discovered that those cysts in her uterus have not been reabsorbed but have turned into a tumor and must be removed immediately. She underwent an operation to remove her ovaries yet now she feels stronger after this experience and has a great desire to become a mother.*

*The repeated confirmation that all is well: monitoring your health.*

This theme emerged from stories in which young women organized the emotional significance of preventive practice as a check on their health and as a periodic confirmation that all is well. This theme, belonging to the category of *affects*, is metaphorically represented by two expressions: to be lifted as if being filled with helium and to stay on the road with an extra file in order and one less pebble in the shoe, fully rendering the sense of relief that monitoring your health can give, as well as the need to take off a worry and continue in a lighter way until further checks.

*The anticipation of the result filled her with anxiety. It was said: it will be negative as usual. But at work, or before falling asleep, every now and then a flash: What if it were positive? Panic. After a few days, she telephoned the studio, her heart pounding. The secretary answered. Giulia: Excuse me, have the results of Giulia Berardi’s pap test arrived? The secretary: Now I’ll check. Yes, everything is OK. Giulia breathed a great sigh of relief.*

*Good information and good health reports: basic elements for preventive examinations.*

This theme emerged from the stories in which young women highlighted the most important elements of prevention: the need to understand, be interested and receive proposals and information in order to decide to undergo a check, as well as the relationship with the doctor, whether a man or a woman. This theme belongs to the category of *thought,* taking up a cognitive level of knowledge.

*A mother, who fears of inheriting the same disease as her mother, undergoes a breast check-up every year; starting from this year, she wants Marta to have a check-up as well. The girl, however, feels embarrassed at the very thought of showing her breasts to the doctor, so her mother suggests that she sees a female doctor. During the test, in which nothing worrying emerges, Marta feels at ease with her. From now on, she will have preventive checks regularly.*

## 4. Discussion

The coding of the stories invented by young women regarding cancer prevention enabled the identification of four theoretical constructs that outline the process of making meaning in the face of the prefiguration of a critical situation, such as that of the early diagnosis of disease or the risk of breast and cervix tumors.

The identification of these aspects provides elements to support the promotion of screening from a young age.

The constructs that emerged—the construction of a defense: youth as protection; the attribution of blame about cancer risk; learning from experience as a prevention activator; and from inaccessibility to access to preventive practices: the creation of engagement—highlight how, in the invention of stories on cancer prevention, young women’s sense making unfolds along a continuum from signification of youth as a protection for oneself and one’s health status without the need for checks and prevention to a reality in which to contemplate the limits and the resources necessary to build one’s own involvement in preventive practices. This is a process that also goes through questions about why a risk of illness should occur, about the guilt of not taking care of one’s self and memories of previous experiences whose coping becomes learning and preventive activation.

The sense-making process with respect to cancer prevention recalls themes that are intertwined with the planes of the body, affects and thought.

In the invention of stories on breast cancer prevention, the expression of strong emotions starting from body injuries and stories of previous illness along the female family line brings, on the one hand, the possibility of making sense and of telling of the experience to act or to prevent; on the other hand, it generates the absence of symbolization and thought in a mental situation of non-containment that requires recourse to concrete thought.

In the invention of stories on the prevention of cervical cancer, what emerges is how much the decision making of prevention involves different levels: the individual plan, with questions that are responsible and inherent in the guilt of not having sufficient self-love; and the relational plan, which contemplates sexuality, the experience of other young people and the relationship with the gynecologist.

This allows us to highlight how the device of the invented story seems to have been constituted as a space capable of guaranteeing young women the appropriate distance from their enemy, which is the risk of cancer and the fear of death, which, owing to their traumatic nature, are normally not representable for the mind. Through the invention of a story, it is possible to talk about unspeakable things, implement a narrative organization and adopt multiple and contradictory points of view [66,67]

With regard to the identification of metaphors, we highlight that 5 of the 12 themes that emerged can be represented through the imaginative use of a metaphor.

In particular, we found a greater presence of metaphors for the theme of *betrayal located in the body and between generations about breast cancer*, assuming that metaphor, with its imaginative and subconscious power, is used to give word to the most difficult object to represent or the anguish of an internal attack on one’s body that can be inscribed in one’s feminine genes as much as it can be derived from a poison produced by what one eats and breathes. Other metaphors concern the possibility of representing the psychic and emotional function of prevention in the theme of repeated confirmation that all is well; therefore, prevention emerges not only as a health request but as a functional practice for one’s own emotions and as a monitoring of one’s state of health. 

In a manner consistent with the literature, we found that metaphors of disease, in its being represented as something casual, refer to a bellicose nature [68] understood by young women as an attack from within that can destroy. However, this does not seem to influence the sense-making process linked to prevention, which metaphorically takes the form of the possibility of monitoring and therefore keeping the anguish of this risk at bay.

Finally, we highlight how metaphors are concentrated more in the categories of body and affects, confirming the fact that metaphors, as structures of thought, help the passage from the bodily and affective level to representability.

Despite the limitations of this study, mainly owing the small sample representative of a single Italian region and the restricted age group, which make it difficult to generalize the results, our work highlights that metaphors make it easier for participants to articulate one’s own experience, both in reference to illness anxiety and preventive action, in line with research suggesting that experience is symbolically encoded [69]. Through the generation of metaphors, young women appear to gain a renewed sense of empowerment with respect to the development of an action plan.

In clinical terms, the articulation of the sense-making process and metaphors by young women provides an in-depth view of their experiences and insights into possible solutions.

## 5. Conclusions

From a psychological and clinical point of view, the results of this work allow us to observe two aspects of particular importance in relation to the construction of an active positioning of subjects towards care for their own health.

First of all, we identified the importance of intercepting the imaginative and therefore prefigurative plan of young women regarding preventive practices for female cancers close to entering the attention and risk range defined by the regional prevention plan. The construction of an internal image of the relationship with the risk of disease and with the possibility of early identification of any of its forms allows us to open up new areas of reflection and to promote a process of prefiguration on the bodily, affective and thought planes invested with respect to this topic. The possibility of managing, handling and making these emotions thinkable through the use of imagination, fantasy and metaphor, as well as through the use of a playful register, makes this process capable of opening a space for internal reflection that is protective of one’s self (the narrator does not coincide with the narrating self). 

Secondly, we think of the possible repercussions in terms of training interventions, awareness raising and dissemination in school and training contexts in terms of spaces for narration and comparison with women who habitually practice prevention or who have gone through the experience of illness or with specialists in the field of screening; this concept can be applied to “modulate” the dominant narrative cultural result that emerged from the stories invented by young women. Knowing, through the invented history, the power of false myths, beliefs, clichés and artifacts transmitted within the culture of belonging offers the opportunity, otherwise difficult to observe, to be able to broaden our sight to a wider context in which screening and self-care are inserted to promote health literacy and active and responsible engagement processes tailored to the culture they belong to.

## Figures and Tables

**Table 1:** Constructs, themes and distribution of metaphors about cancer prevention in invented stories.

	The Construction of a Defense: Youth as Protection	The Attribution of Blame about Cancer Risk	Learning from Experience as a Prevention Activator	From Inaccessibility to Access to Preventive Practices: the Creation of Engagement
**BODY**	*Health is invisible and silent; illness makes noise and gives signals.* 1 Metaphor: Disease as extraction of a number or a name.	*A betrayal located in the body and between generations: breast cancer* 3 Metaphors: -The laceration of the body attacked and filled with poison. -The uncontrollable invader. -Breast and uterus were twins, and when one got angry, the other tried to reassure it by passing its energy and strength to it.	*Scars and losses that teach*	*Prevention that creates irreparable damage*
**AFFECTS**	*Being invincible: it can’t happen to me.*	*A deceptive and punitive sexuality: cervical cancer*	*Stories of illnesses (own or of others) to be transformed into active* 1 Metaphor: The waiting room emerges as a merciless purgatory.	*The repeated confirmation that all is well: monitoring your health* 2 Metaphors: -To be lifted as if being filled with helium. -To stay on the road with an extra file in order and one less pebble in the shoe
**THOUGHT**	*Self-care is the aesthetic care of your body*	*The difficult transmission of love and self-knowledge* 1 Metaphor. Being a woman who stops at the zip of her jeans.	*The warning of a false alarm or an accident on the way*	*Good information and good health reports: basic elements for preventive examinations*

## Data Availability

The data are available upon request to the corresponding author.
